# Bayesian Geostatistical Modeling to Assess Malaria Seasonality and Monthly Incidence Risk in Eswatini

**DOI:** 10.1007/s44197-022-00054-4

**Published:** 2022-08-17

**Authors:** Sabelo Nick Dlamini, Ibrahima Socé Fall, Sizwe Doctor Mabaso

**Affiliations:** 1 Department of Geography, University of Eswatini, Kwaluseni, Manzini, M200 Eswatini; 2 World Health Organization, 27 Geneva, Geneva, Switzerland

**Keywords:** Malaria, Bayesian modeling, Geostatistics, Distributed lag model, Eswatini

## Abstract

Eswatini is on the brink of malaria elimination and had however, had to shift its target year to eliminate malaria on several occasions since 2015 as the country struggled to achieve its zero malaria goal. We conducted a Bayesian geostatistical modeling study using malaria case data. A Bayesian distributed lags model (DLM) was implemented to assess the effects of seasonality on cases. A second Bayesian model based on polynomial distributed lags was implemented on the dataset to improve understanding of the lag effect of environmental factors on cases. Results showed that malaria increased during the dry season with proportion 0.051 compared to the rainy season with proportion 0.047 while rainfall of the preceding month (Lag2) had negative effect on malaria as it decreased by proportion − 0.25 (BCI: − 0.46, − 0.05). Night temperatures of the preceding first and second month were significantly associated with increased malaria in the following proportions: at Lag1 0.53 (BCI: 0.23, 0.84) and at Lag2 0.26 (BCI: 0.01, 0.51). Seasonality was an important predictor of malaria with proportion 0.72 (BCI: 0.40, 0.98). High malaria rates were identified for the months of July to October, moderate rates in the months of November to February and low rates in the months of March to June. The maps produced support-targeted malaria control interventions. The Bayesian geostatistical models could be extended for short-term and long-term forecasting of malaria supporting-targeted response both in space and time for effective elimination.

## Introduction

Malaria transmission has until recently continued to decline in Southern Africa thus encouraging national control programs in the region to focus on elimination [1, 2]. However due to disruptions during the COVID-19 pandemic, the World Health Organization (WHO) globally reported 241 million cases in 2020, a slight increase from those reported in 2019 which stood at 227 million. A total of 627 000 deaths were attributed to malaria in 2020 compared to about 558 000 in 2019 [3]. About 93% of these cases and deaths occur in sub-Saharan Africa. Interestingly, in the same year 2020, about 26 countries reported fewer than 100 indigenous cases of the disease, an increase from 6 countries in 2000. To be eligible for WHO certification as malaria free, countries must achieve at least 3 consecutive years of zero indigenous cases. Since 2015, about 9 countries have been certified by the WHO Director-General as malaria-free and they include Maldives (2015), Sri Lanka (2016), Kyrgyzstan (2016), Paraguay (2018), Uzbekistan (2018), Argentina (2019), Algeria (2019), China (2021) and El Salvador (2021) [4].

Eswatini (formerly known by its English name as Swaziland), is a country on the brink of elimination and had however had to shift its target year on several occasions since 2015 due to persistent cases that continued to be reported albeit sporadic in some parts of the country. Ever since 2015, the country had been setting new targets each year as it struggled to maintain zero cases. Recently, in 2019, Eswatini launched a domestic malaria fund to strengthen efforts towards its elimination initiative (Eswatini Government, 2019). Reaching and maintaining zero cases had been a challenge for most countries including Eswatini [5]. Eswatini with a population of 1.16 million had seen a fluctuation in malaria cases with a high degree of uncertainty as there were 460 cases in 2012 and rising to a peak of 1198 cases in 2017 while 693 were reported in 2019 [6].

Indeed, with recent domestic funding initiatives the country has demonstrated its political will to unrelentingly push towards malaria elimination. Such political will requires scientific evidence to guide the path towards elimination in terms of both the necessary strategies and tools [7]. Consequently, strong surveillance and sustained control intervention strategies are needed in the critical phase prior to elimination [8, 9]. Bayesian geostatistical models using environmental covariates had been applied in malaria mapping in various endemic settings and countries [10–13]. In addition, distributed lag models (DLMs) are ideal for estimating epidemic build-up whereby certain weather conditions and elapsed time before onset of epidemics could be estimated [14, 15]. The application of DLMs has a potential to support control programmes in timely deployment of control interventions, thereby effectively aid malaria surveillance and control efforts [16–18].

Eswatini had not only rebranded itself from a National Malaria Control Programme (NMCP) to a National Malaria Programme (NMP) but according to [19] has already halted endemic transmission and is currently relentlessly pursuing elimination [20]. Nonetheless, recent malaria cases trend showed that the country would likely continue to struggle with bringing cases to zero especially due to the ever-present threat of importation from neighboring regions adding to seasonal case load uncertainties. For instance, a study by [21] showed that importation from Mozambique accounted for over 90% of malaria transmission in Eswatini, thus retarding ongoing control efforts. Furthermore, data from the Eswatini Malaria Programme continued to show unpredictable seasonal fluctuations in cases. Such seasonal fluctuations and upsurges in cases reemphasize the need for stronger surveillance systems and watchfulness even when endemic transmission had been halted [19, 21].

Eswatini has its historic malaria transmission occurring in the eastern part of the country where until recently low unstable transmission characterizes the malaria situation in the area [22]. Its seasonal peaks had been associated with episodes of high rainfall during the summer season which occurs between November and May each year [23]. We applied Bayesian geostatistical modeling [24] to predict heightened transmission seasons by quantifying the elapsed time prior to onset of cases. The elapsed time refers to the amount of time that passes from the start of an event to its finish and in this case it refers to the cumulative environmental conditions that result to reported local cases. Geostatistical models link the disease data with potential environmental predictors and quantify spatial dependence via the covariance matrix of Gaussian process facilitated by adding random effects at the observed locations [25]. Knowledge of the elapsed time and space location of cases could aid the Malaria Programme to accurately deploy malaria prevention measures in advance. Furthermore, these models could be used as tools to guide both primary and secondary response measures i.e. prior to onset of cases by estimating the elapsed time and after onset by predicting malaria risk thus assisting malaria programmes to prevent onward transmission.

Spatially explicit model-based maps on micro epidemiological heterogeneities are important for malaria elimination as endemic transmission declines to residual foci [26]. These maps aid surveillance and vector control efforts in better targeting and in deployment of planned interventions [27, 28]. In this study, Eswatini malaria incidence data were fitted into a Bayesian geostatistical negative binomial model using a polynomial distributed lag function. We chose DLMs because they are useful when the outcome of interest is a result of a cumulative effect from previous time periods [29]. The DLM function can be used to assess if the effect of risk factors on the outcome is either immediate or rather slowly as a result of a build up from previous climatic conditions. We then produced smoothed maps of incidence risk during rainy and dry season as well as monthly risk maps for the entire country.

## Methods

### Study area

Eswatini is a country located in southern Africa, specifically in the north eastern part of South Africa and close to the southern part of the Mozambican border by about 90 km. The country is 100% landlocked, whereby over 90% of its borders are shared with South Africa while the north-eastern side is bordered by Mozambique [30]. Eswatini is a developing country and is about 70% rural and a majority of its rural folks derive their livelihoods from substance agriculture. Those that reside in urban centers and towns are mostly sustained by formal employment and small to medium formal and informal businesses. In Eswatini, malaria transmission is seasonal and dependent on the prevailing weather conditions especially with regards to temperature and rainfall [31]. The local malaria epidemiology also shows that malaria transmission is highly affected by variations in altitude [32]. The country is comprised of four agro-ecological zones which vary by altitude, climate, soil type and vegetation. The variations in the agro-ecological zones have also influenced population distribution and settlement within the country.

### Malaria Incidence Data

**Fig. 1:**
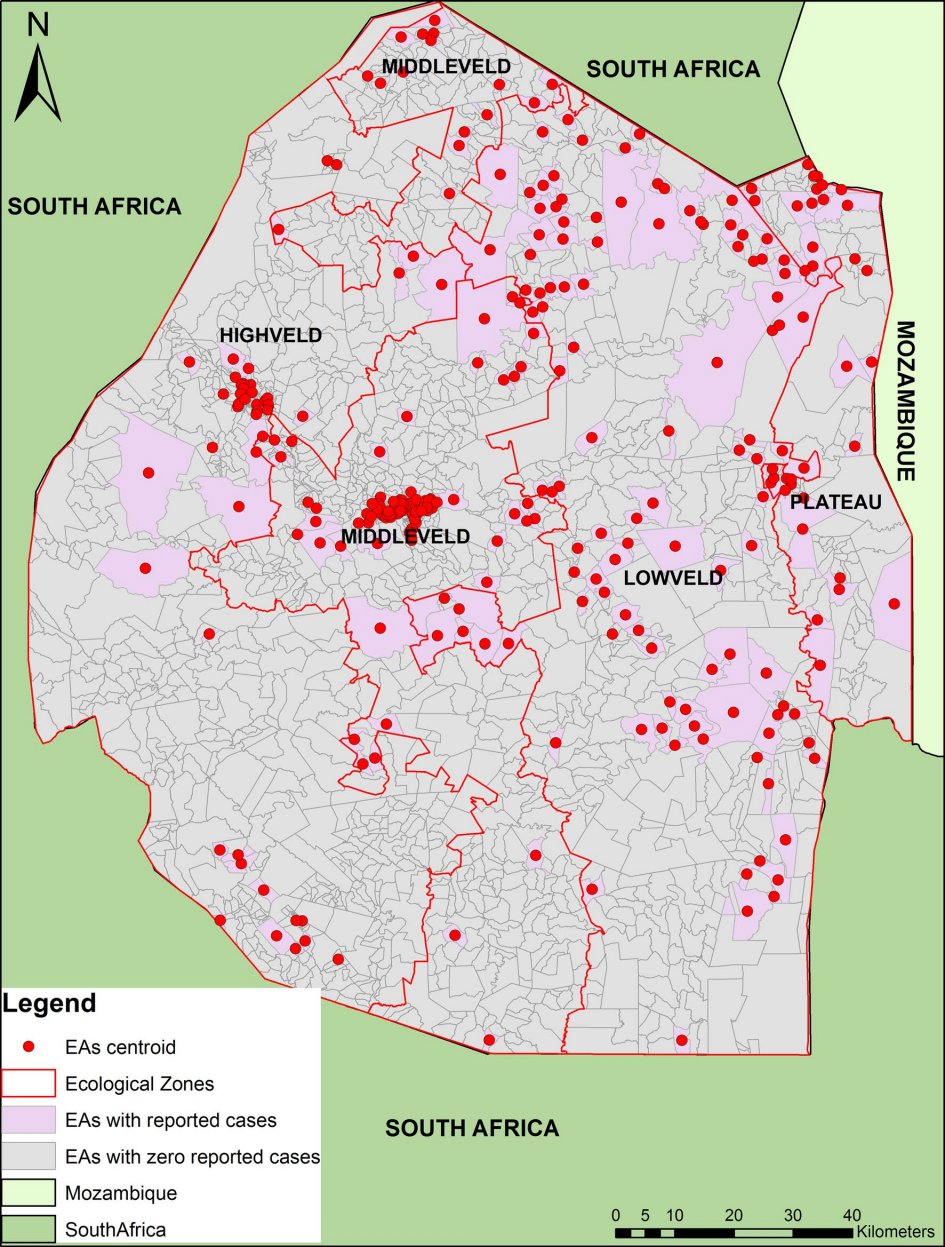
Study area showing enumeration areas with reported cases

Georeferenced malaria incidence data for a 5 year period (2012–2017) were obtained from the National Malaria Programme (NMP) of Eswatini. The data comprised of reactively investigated symptomatic cases that have presented at health facility. Cases were already classified into either imported or local based on travel history of patients by investigating officers from the NMP. Local cases with valid geographic coordinates were aggregated by enumeration area (EA) which is the lowest census unit ranging from an area of about 0.013 km^2^ to about 194 km^2^. The country is made up of a total of 2326 EAs and only those EAs with reported cases were used during analysis (Fig. 1). In addition, cases identified via active case detection in the neighboring households of an index case were also included in the analysis. The population in each of the EAs was used as an offset in the negative binomial model to implement weighting by number of people. The data were organized according to the malaria transmission season which is July to June each year. A total of 1230 georeferenced malaria cases, both local and imported were used in the analysis. Local malaria cases were reported in 529 household locations which were aggregated into 145 EAs taking into account the time resolution. Reported georeferenced cases were analysed using ArcGIS software version 10.8.1 and STATA statistical software version 13 [33]. Bayesian modeling was done using OpenBUGS software, copyright (C) 2007 Free Software Foundation, Inc. (http://fsf.org/).

### Environmental Data

Remotely sensed climatic data covariates were downloaded from NASA earth data website (https://search.earthdata.nasa.gov/). These data included a 250 m resolution normalized difference vegetation indices (NDVI) available biweekly and 250 m day and night land surface temperature (LST) emissivity indices both available weekly. The LST was added as LST day and LST night in the modeling to capture the effect of day and night temperatures on malaria cases. The data were products of the Moderate Resolution Imaging Spectroradiometer (MODIS). Eight km resolution dekadal rainfall was obtained from the Africa Data Dissemination Service (ADDS), a data portal for the Famine Early Warning Systems (FEWS) network. In addition, a 30 m resolution Digital Elevation Model (DEM) from ASTER (Advanced Spaceborne Thermal Emission and Reflection Radiometer) was obtained for Eswatini. Water bodies were digitized from a 2016 Google Earth Image. The environmental data used are presented in Table 1 below.

**Table 1:** Environmental data analysed

Factors	Spatial resolution	Temporal resolution	Period	Source
NDVI	250 m	16 days	2012–2016	MODIS
LST	250 m	8 days	2012–2016	MODIS
Rainfall	8 km	Dekadal	2012–2016	ADDS
Water bodies	30 m	Yearly	2016	Google earth
Altitude	30 m	Yearly	−	ASTER
Population	−	−	2017	Eswatini census

### Fitting a Distributed Lag Model

We formulated and implemented a Bayesian distributed lag model (DLM) to better understand the association between certain environmental conditions favourable to malaria transmission (i.e. precipitation, temperature and vegetation) and an increased number of malaria reported cases at lag of 0, 1 or 2 months to account for previous conditions prior to malaria incidence as well as quantify the elapsed time (lag) prior to onset of malaria reported cases. Other factors included consisted of fixed terms (i.e. altitude and distance to water bodies) in the following fashion:

Let $Y_{it}$ be the number of malaria cases reported at location $i$ during month $t$, modelled using a Negative Binomial distribution to take into account the overdispersion of the counts as cases in the country were already diminishing:

1 $$Y_{it} \sim NBin\left( {p_{it} ,r_{i} } \right)\;\;\,\forall i = 1,.....,n\,\;\;\forall t = 1,....,T$$

where $T=60 \mathrm{months for the }5\mathrm{ year period}$ and ${ p}_{it}={r}_{i}/({r}_{i}+{\mu }_{it})$. The parameter ${\mu }_{it}$ represents the mean counts and ${r}_{i}$ is the overdispersion parameter. The regression equation that links the mean counts to the space and time variables takes the form of a log-linear equation:

2 $$\begin{aligned} \log \left( {\mu _{{it}} } \right) =\,& \log \left( {{\text{pop}}_{{it}} } \right) + \mathop \sum \limits_{{l = 0}}^{L} \alpha _{l} {\text{Rainfall}}_{{i,t - l}} \\ &+ \mathop \sum \limits_{{l = 0}}^{L} \beta _{l} {\text{DayLST}}_{{i,t - l}} + \mathop \sum \limits_{{l = 0}}^{L} \gamma _{l} {\text{NightLST}}_{{i,t - l}}\\ & + \mathop \sum \limits_{{l = 0}}^{L} \delta _{l} {\text{NDVI}}_{{i,t - l}} + \varphi ~{\text{Altitude}}_{i} \\ & + \tau ~{\text{Distance\_Water}}_{i} \\ &+ \varepsilon ~{\text{Season}}_{t} + \zeta ~{\text{Imported}}_{{it}} + ~\omega _{{ea\left( i \right)}} \end{aligned}$$

where ${Season}_{t}$ is a binary variable indicating whether the case was reported during rainy season (November to April) or dry season (May to October) and ${Imported}_{it}$ is a a binary variable representing the presence of at least one case classified as imported in the same EA in the preceding 2 months. It is important to note that here we assume the seasonal pattern that characterize malaria transmission not to change through the years. To take into account the spatial correlation between locations, we add a Gaussian spatial process ${\omega }_{ea\left(i\right)}\sim N\left(0, \Sigma \right)$ where the element $(ij)$ of the variance–covariance matrix for two locations at distance ${d}_{ij}$ is of the form${\Sigma }_{ij}={\sigma }^{2}\mathrm{exp}\left(-\rho {d}_{ij}\right)$. We indicate with ${\sigma }^{2}$ the spatial variance and with $\rho$ the correlation decay. We need to specify prior distributions for the parameters we want to estimate, since we are specifying the model under a Bayesian framework. We assign uninformative priors on the coefficients$\varphi , \tau$,$\varepsilon , \zeta$ such as $N(\mathrm{0,0.01})$. The coefficients identifying the lagged effect need to be constrained ${\alpha }_{l} \sim N\left(0,{\sigma }_{Rainfall}\right),$ ${\beta }_{l} \sim N\left(0,{\sigma }_{DayLST}\right) ,$ ${\gamma }_{l} \sim N\left(0,{\sigma }_{NightLST}\right),$ ${\delta }_{l} \sim N\left(0,{\sigma }_{NDVI}\right) \forall l=0,\dots ,L$. Inverse-gamma priors were assumed for the variances.

### Fitting a Polynomial Distributed Lag Model

A second Bayesian model based on polynomial distributed lags was again formulated and implemented on the same dataset to improve understanding of the lag effect of environmental factors on the number of malaria cases at distributed lags of up to two months (0,1 and 2 months). Data were aggregated bi-weekly (using full calendar weeks for each month which generated16 days per lag) up to two months. This was done to determine the best combination of lags that predicted malaria incidence risk. The data was then fitted into the Negative Binomial model with a polynomial function constrained to power ${x}^{4}$ in the following fashion:

Let ${Y}_{it}$ be the mean number of malaria cases at a given location *s* = $i$,….$n$ at time $t$ with likelihood ${Y}_{it}$ ~$NBin$ (${P}_{i}$, $r$) where ${P}_{it}$ is the proportion of malaria cases in a defined location in time and $r$ is the overdispersion parameter and ${\mu }_{it}=r\frac{1-p}{p}$ while ${\sigma }_{it}^{2}=r(1-p){p}^{-2}$. The model then takes a log-linear equation as:

3 $$Logit\left( {\mu_{it} } \right) = \log it\left( {Popu_{i} } \right) + \beta_{0} + \beta_{1} X_{it} \ldots ..\beta_{12} X_{it} + \varepsilon_{{it + \omega_{ea\left( i \right)} }}$$

where ${\mu }_{it}$ is the number of malaria cases in each location $i$ at bi-week time $t$ and $\beta$ are the regression coefficients, $X$ are the model covariates, $\varepsilon$ and $\omega$ are temporal (bi-week) and spatial random effects (EA). The individual β coefficients of the distributed lag model were restricted to a polynomial function of ${x}^{4}$ which was specified as:

4 $$\beta_{i} = \mathop \sum \limits_{k = 0}^{4} a_{k} i^{k}$$

where $k$ is the categorical variable for the covariate corresponding to ${\beta }_{i}$ coefficient and $a$ is the intercept for locations $1\dots .n$. The model describes the association between an independent value of ${X}_{i}$ and the corresponding dependent mean ${Y}_{i}$. This is summarized as$E\left( {y\left| x \right.} \right)$. The model gives the expected ${\mu }_{i}$ of malaria cases given the corresponding value of each categorical variable at location *s*.

### Determining Important Lags Using Bayesian Variable Selection

We applied Bayesian variable selection to determine the most important lag time between environmental factors and the onset of malaria cases. We used a 95% Bayesian credible interval to find those independent variables that were significantly associated with the outcome variable of interest thus allowing us to fit the model only for those variables that were significant in the final model. The set of $${\beta }_{i}$$ predictors were fixed into a polynomial function describing the distribution of each set of predictors where the third power $${(x}^{4})$$ was selected following first stage testing of the different polynomials (i.e. from $${x}^{1}$$ to $${x}^{7}).$$ The model was then restricted to $${x}^{4}$$ for all the predictors comprising of LST, NDVI and Rainfall.

For each of the polynomial functions ${x}_{i}$ we introduced a binary indicator with 50% chance of inclusion into the final model by restricting the variable selection to a Bernoulli distribution [34] with probability of inclusion whereby the best set of covariates was indicated by the model with the highest posterior probability ranging from 0 to 1. Any variable with coefficient above 50% was selected to the final model. To enable prediction, we used an inverse gamma prior and thenwe ran the model using Markov Chain Monte Carlo (MCMC) [35]. Uninformative prior distributions were also assigned to the parameters to complete the model formulation. We then applied Bayesian kriging [36] to predict the malaria incidence risk at unsampled locations and produced monthly malaria incidence risk maps of the entire country. The input point count in the kriging for the fixed count search was set at 10 points while the search radius was 50 m to identify clusters associated with local malaria cases as mosquitoes are believed to fly a minimum of 50 m [37]. This way we were able to identify months with low, moderate and high transmission periods. To validate the model, we used a randomly selected sample of 150 locations as a training set for fitting the final prediction model.

## Results

### Estimated Parameters Of The Fitted DLM Model

**Fig. 2:**
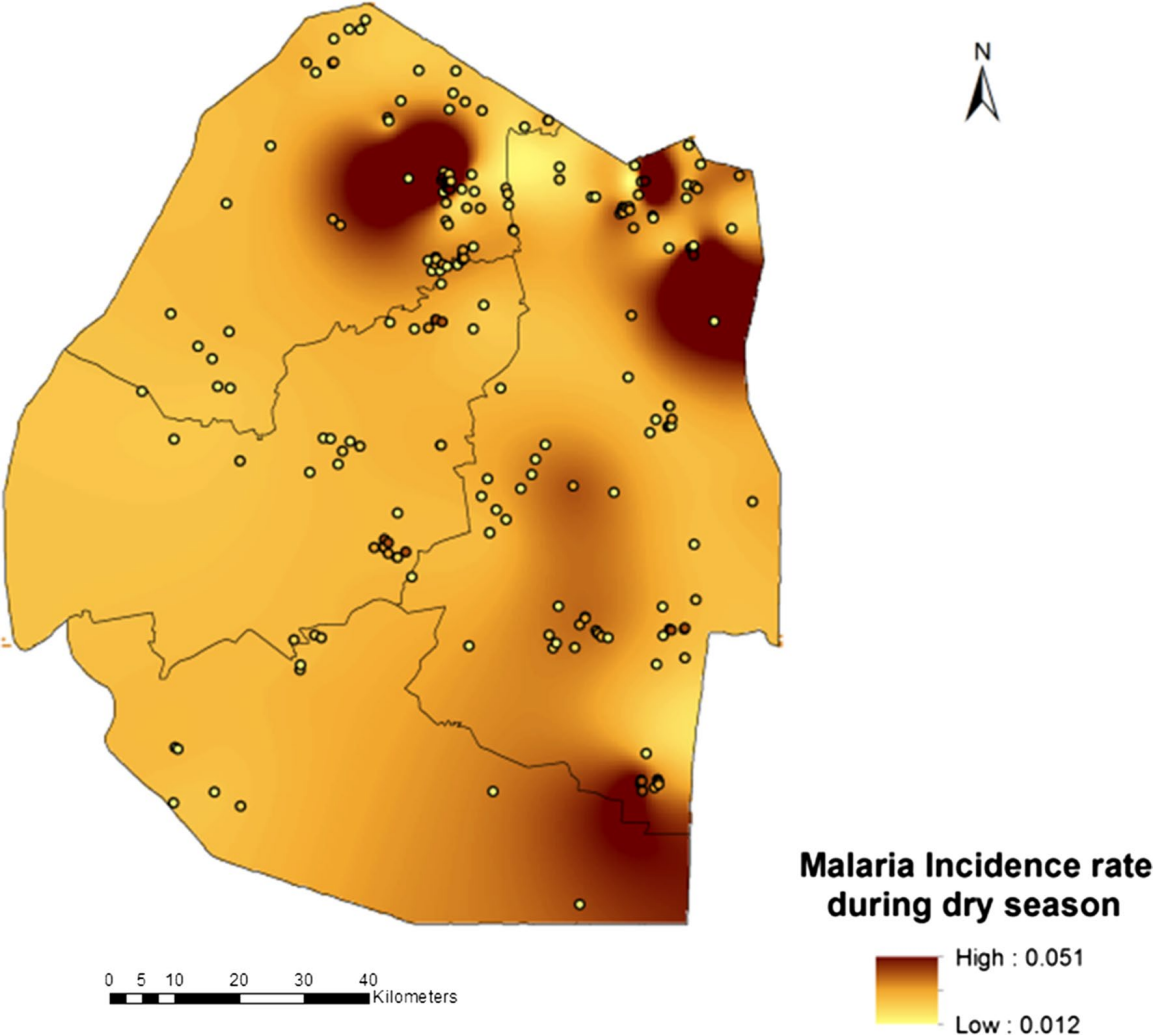
Malaria incidence during dry season

**Fig. 3:**
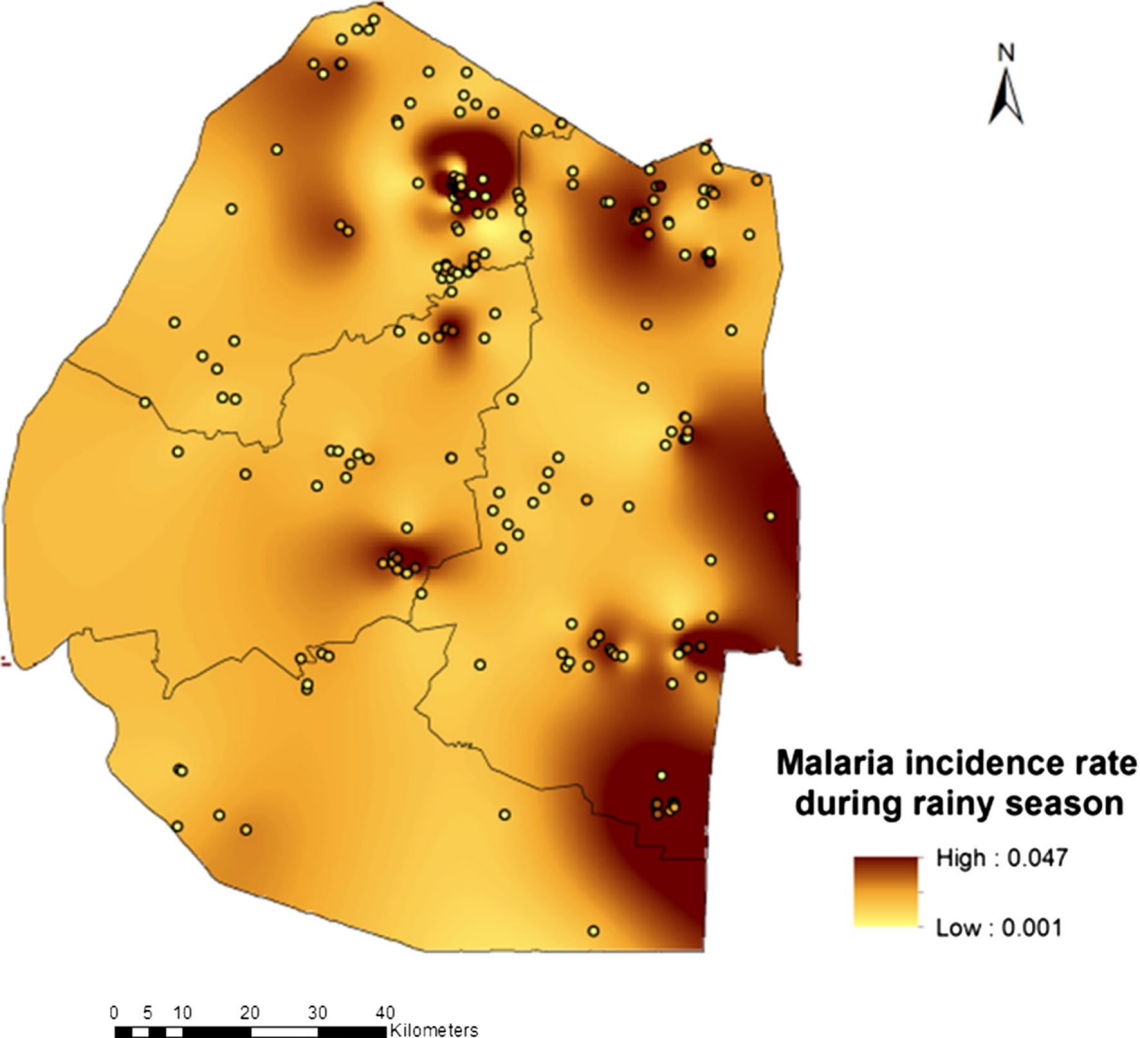
Malaria incidence during rainy season

Results showed that rainfall of the preceding month (Lag2) had negative effect on malaria incidence rate as it decreased by proportion − 0.25 (BCI: − 0.46, − 0.05). This was also shown in the accompanying maps (Fig. 2) which showed that malaria incidence increased during the dry season by proportion 0.051 (5.1%) compared to the wet season where it was 0.047 (4.7%) (Fig. 3). Interestingly the night temperatures of the preceding first and second month were also significantly associated with increased malaria incidence rate at proportions: Lag1 0.53 (BCI: 0.23, 0.84) and at Lag2 0.26 (BCI: 0.01, 0.51). In addition, seasonality was also important predictor of malaria incidence rate with proportion 0.72 (BCI: 0.40, 0.98). Other predictors such as NDVI, LSTday, Distance to Water bodies, Altitude as well as Imported cases were not significant. However, it is important to note that except for NDVI and LSTday which were fitted into the distributed lag model the rest were fixed parameters. Table 2 presents the results of the model predictors adjusted for seasonality and the effects of importation on malaria incidence rate.

**Table 2:** Results of fitted distributed lag model

Covariates	Lag0 Median (95% CI)	Lag1 Median (95% CI)	Lag2 Median (95% CI)	Median (95% CI)
Rainfall	− 0.14 (− 0.35, 0.06)	− 0.21 (− 0.42, 0.00)	− 0.25 (− 0.46, − 0.05)	–
LSTday	− 0.21 (− 0.53, 0.09)	− 0.03 (− 0.35, 0.28)	− 0.12 (− 0.40, 0.14)	–
LSTnight	0.24 (− 0.08, 0.57)	0.53 (0.23, 0.84)	0.26 (0.01, 0.51)	–
NDVI	− 0.14 ( -0.49, 0.20)	− 0.11 (− 0.59, 0.37)	0.13 (− 0.19, 0.46)	–
Altitude	–	–	–	0.04 (− 0.13, 0.21)
Distance to Water bodies	–	–	–	0.02 (− 0.12, 0.16)
Season	–	–	–	0.72 (0.40, 0.98)
Imported	–	–	–	− 0.11 (− 0.63, 0.41)

### Estimated Parameters Of The Fitted Polynomial DLM Model

**Fig. 4:**
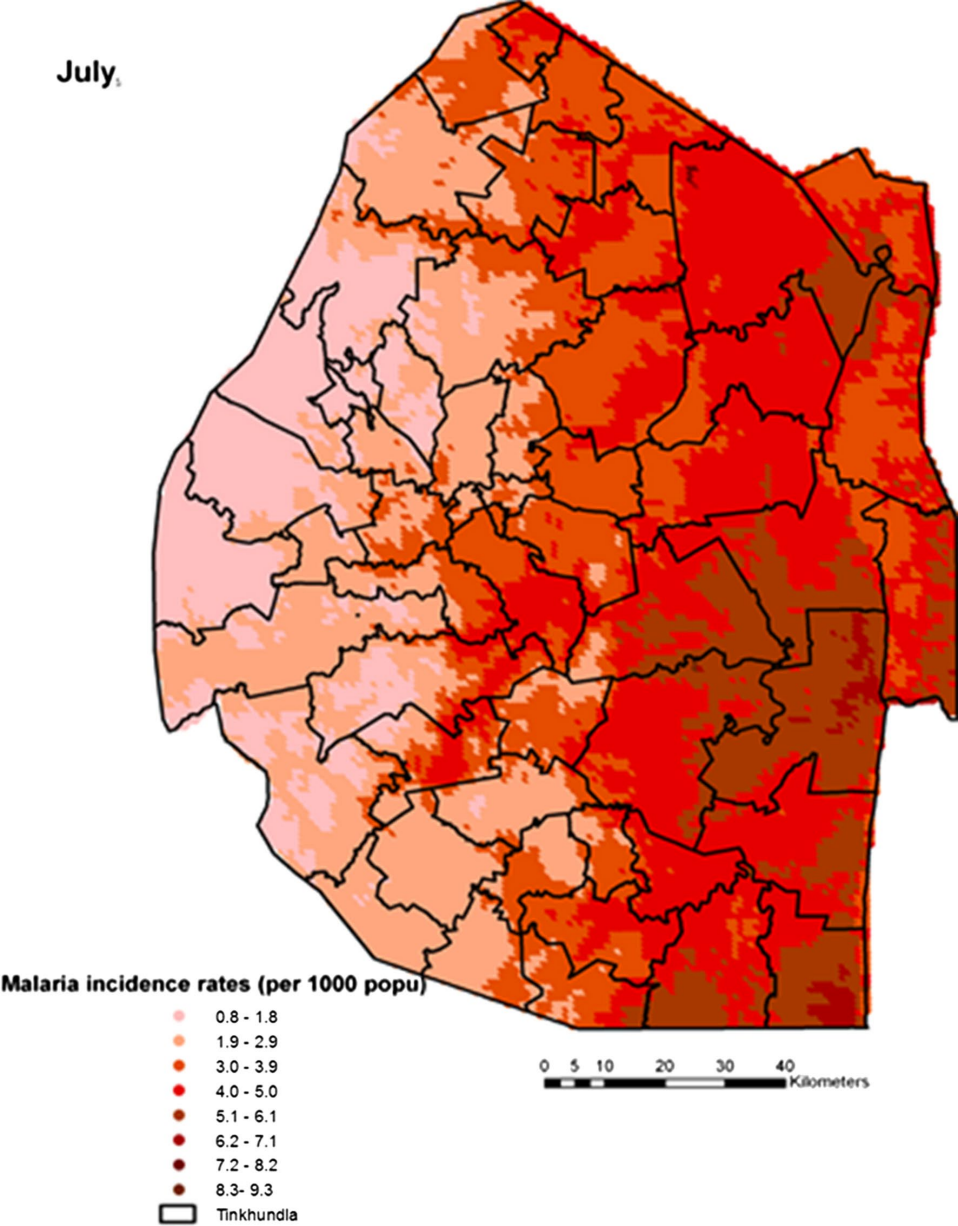
Malaria incidence during the month of July

**Fig. 5:**
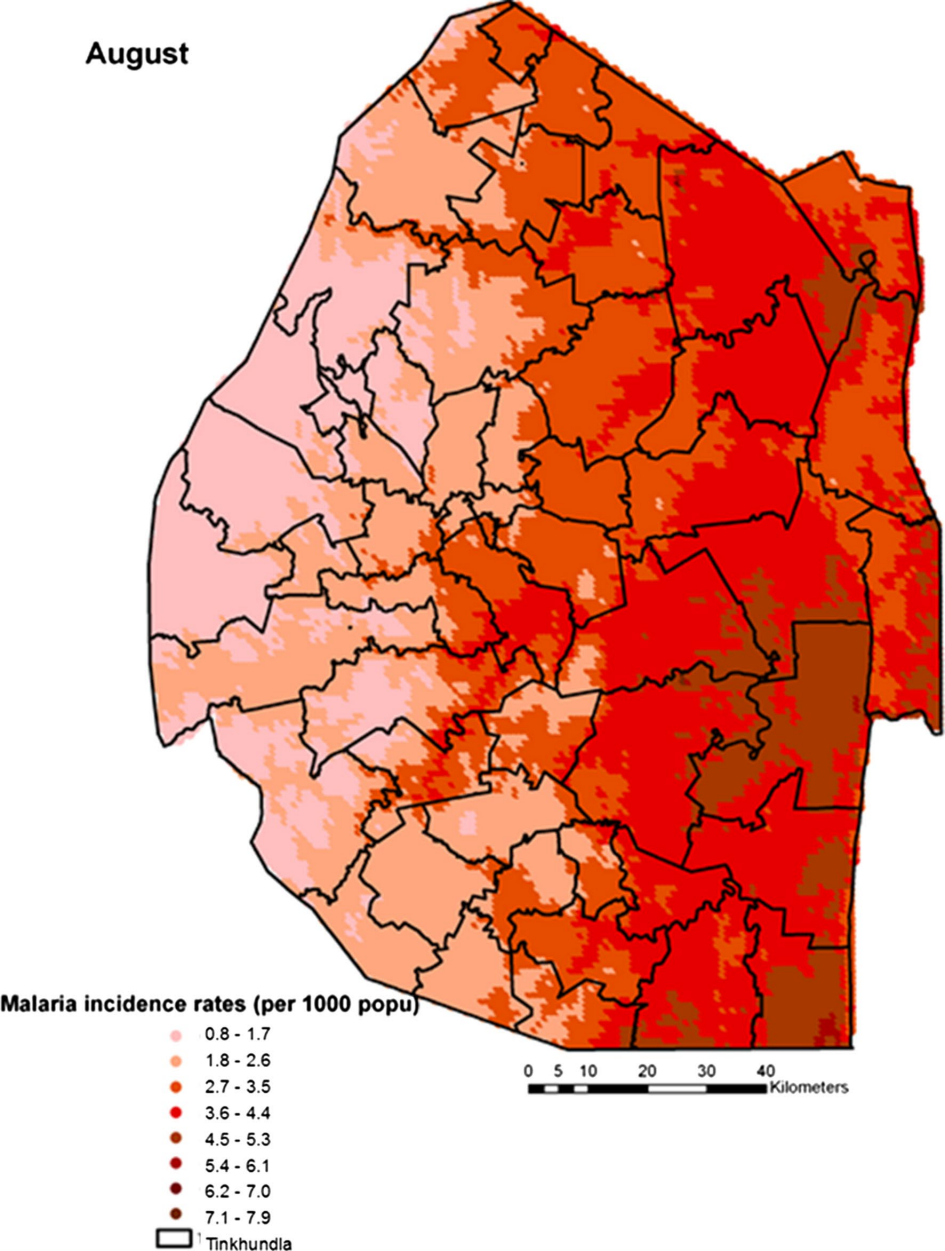
Malaria incidence during the month of August

**Fig. 6:**
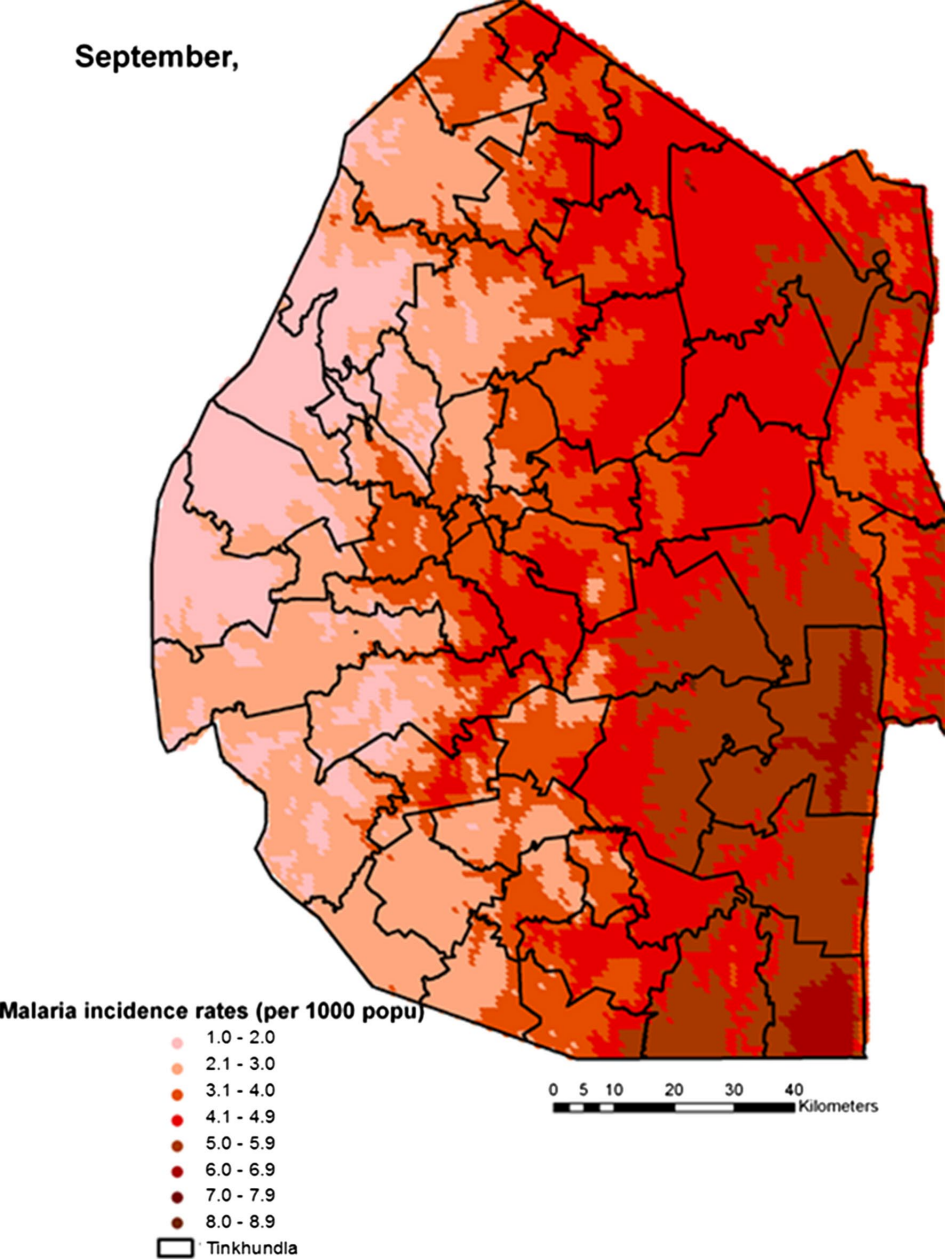
Malaria incidence during the month of September

**Fig. 7:**
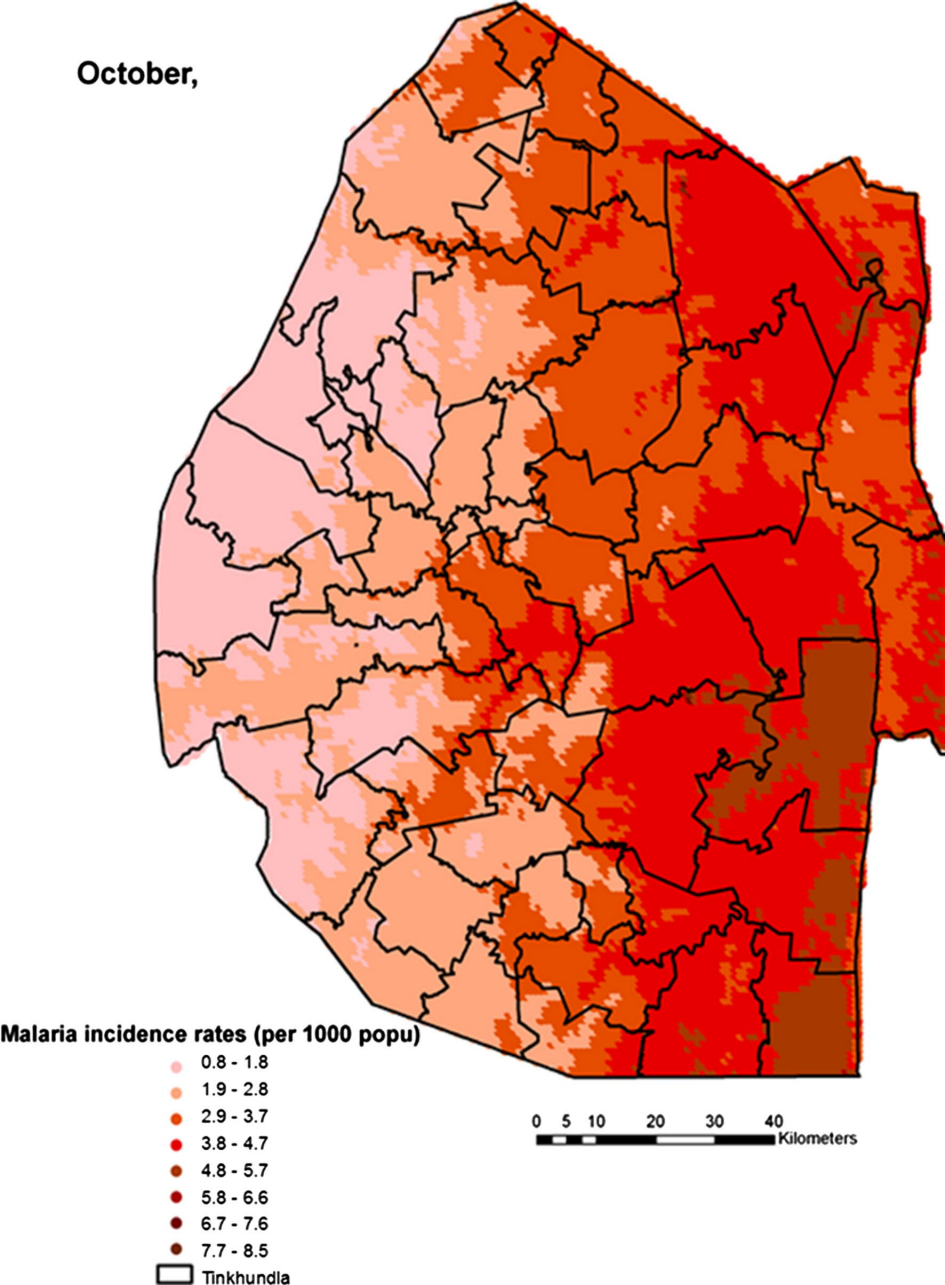
Malaria incidence during the month of October

**Fig. 8:**
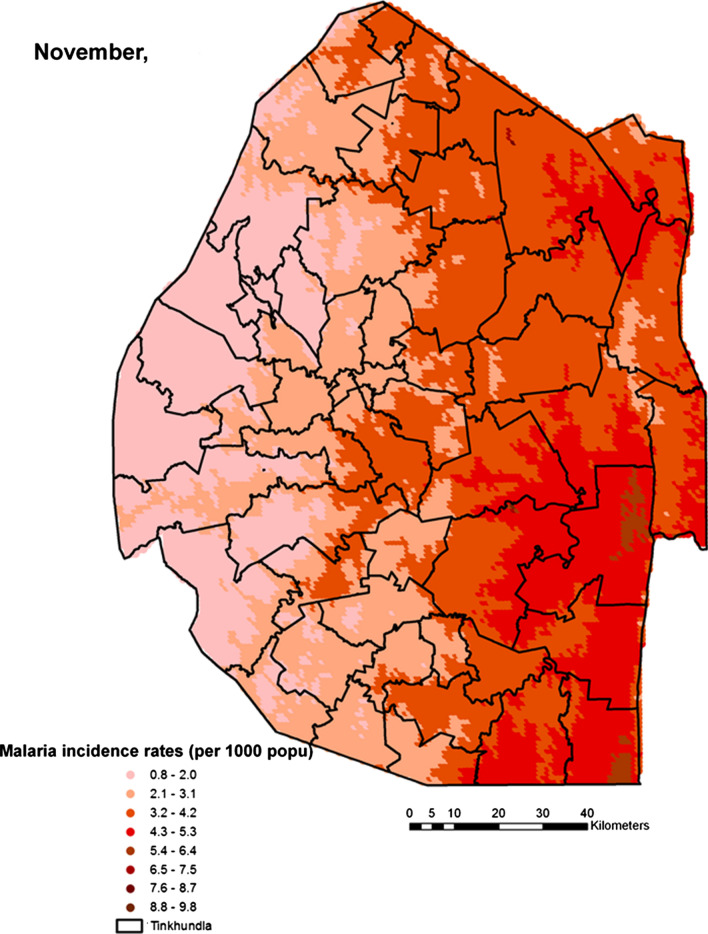
Malaria incidence during the month of November

**Fig. 9:**
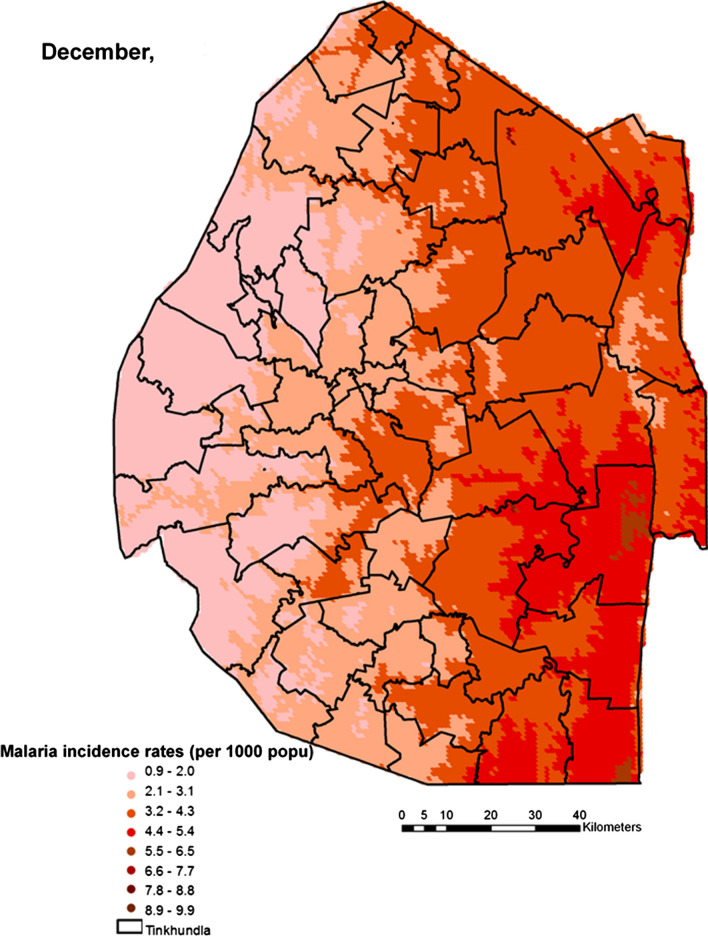
Malaria incidence during the month of December

**Fig. 10:**
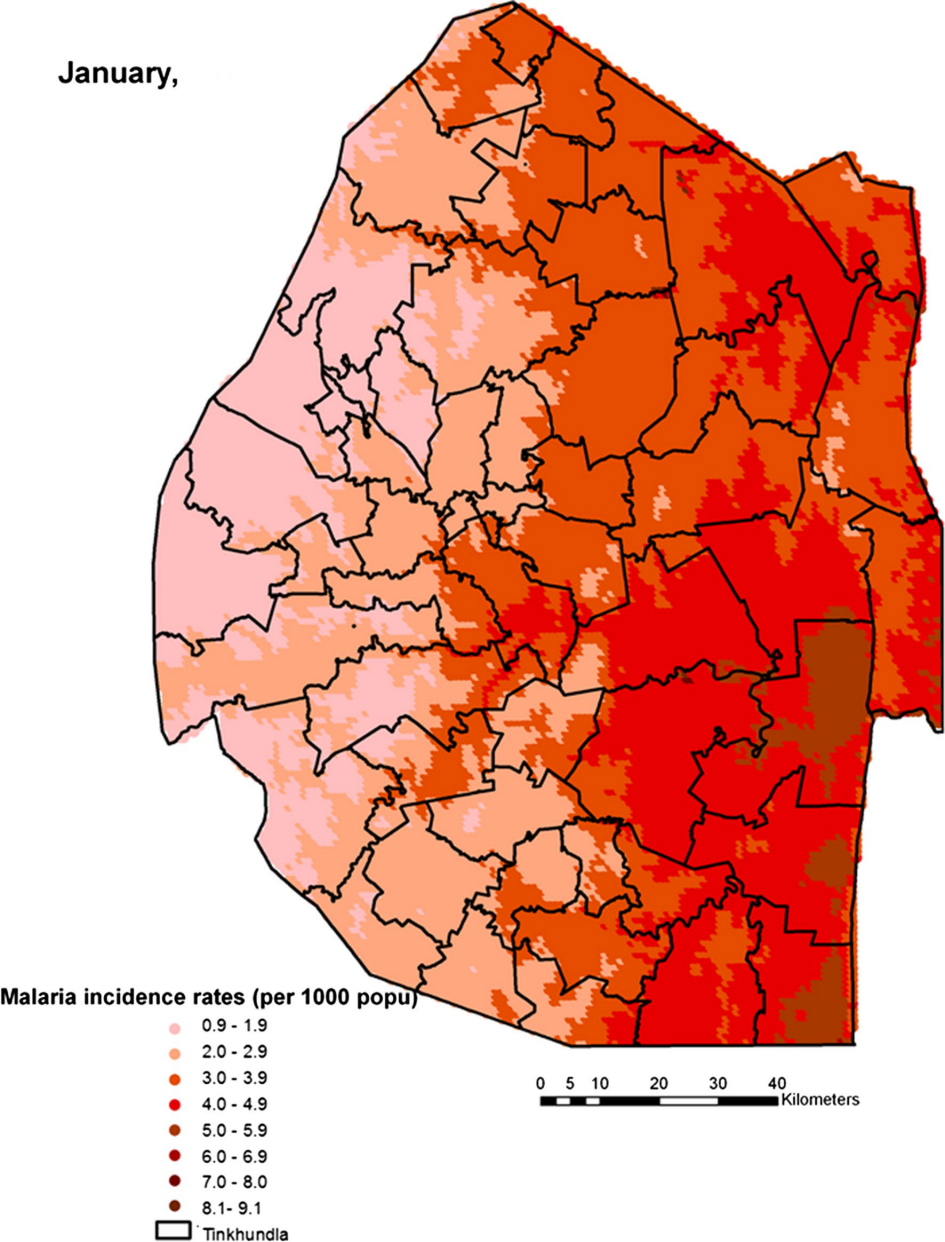
Malaria incidence during the month of January

**Fig. 11:**
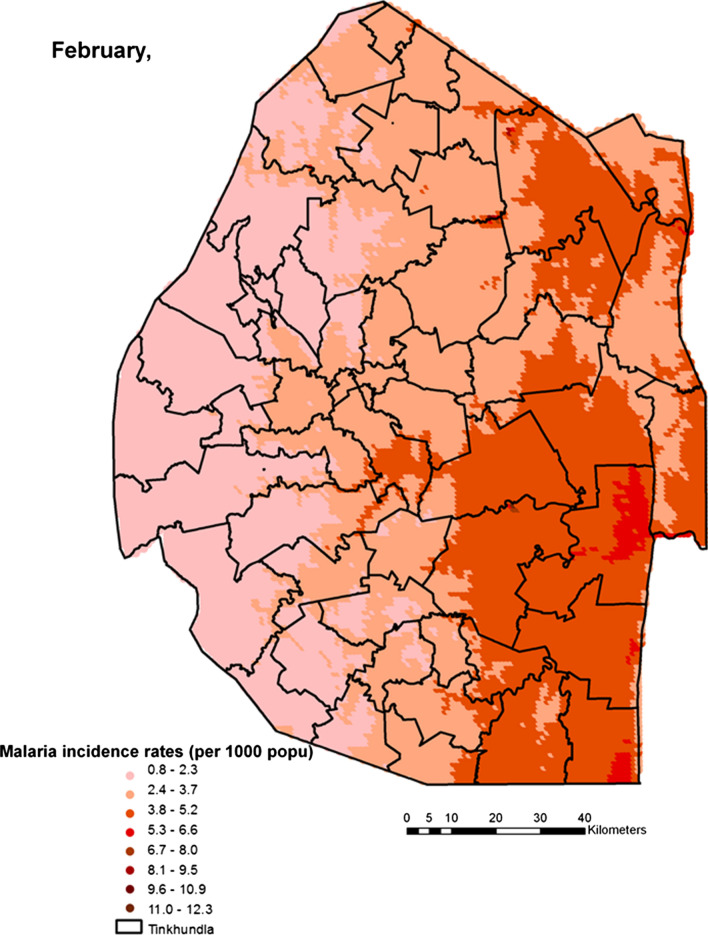
Malaria incidence during the month of February

**Fig. 12:**
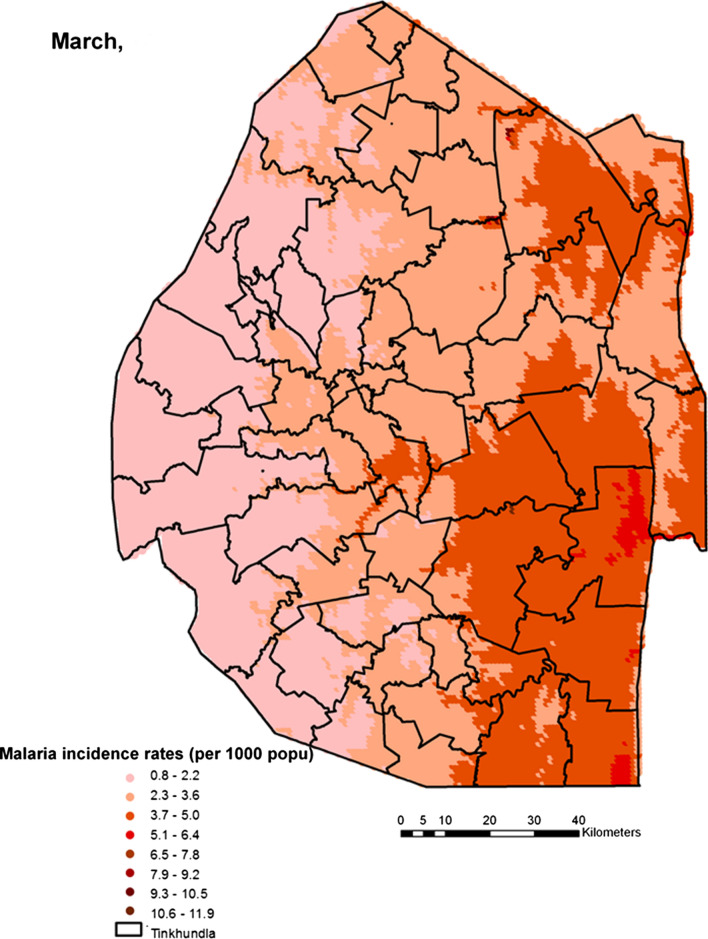
Malaria incidence during the month of March

**Fig. 13:**
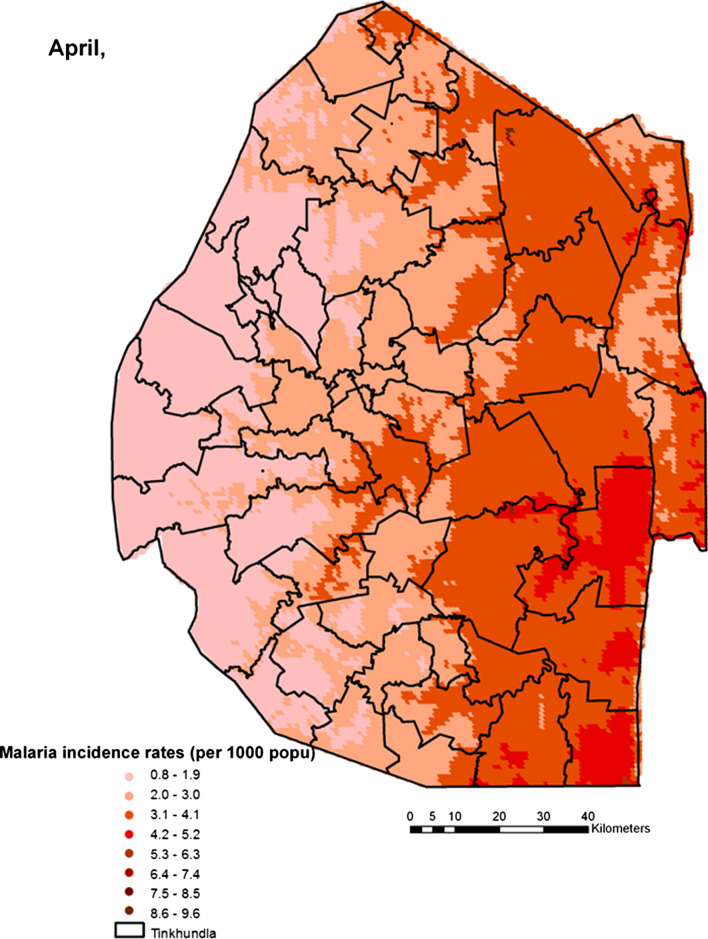
Malaria incidence during the month of April

**Fig. 14:**
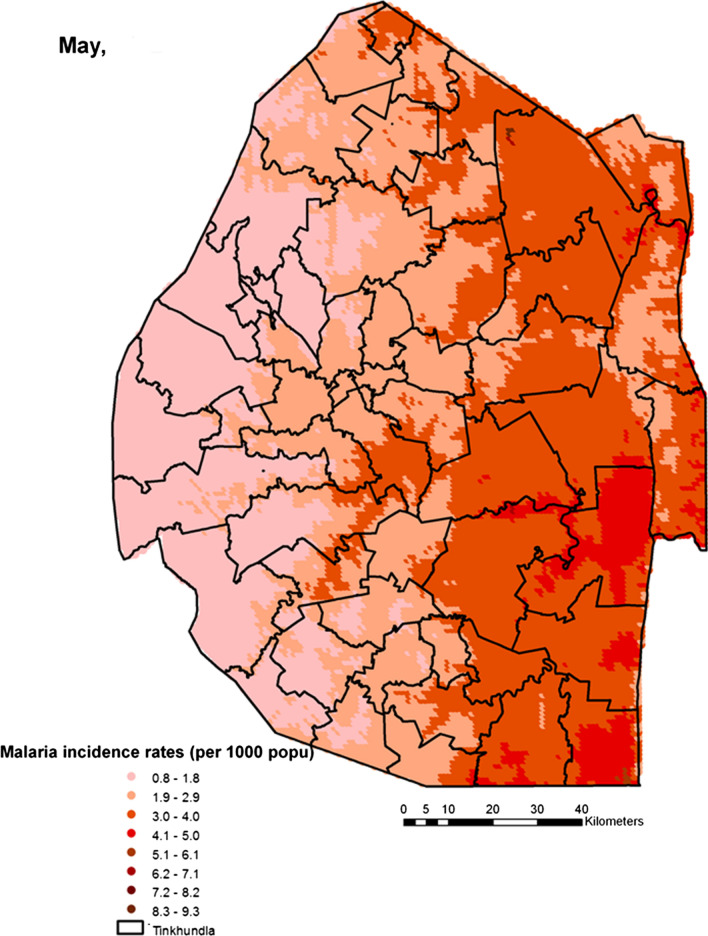
Malaria incidence during the month of May

**Fig. 15:**
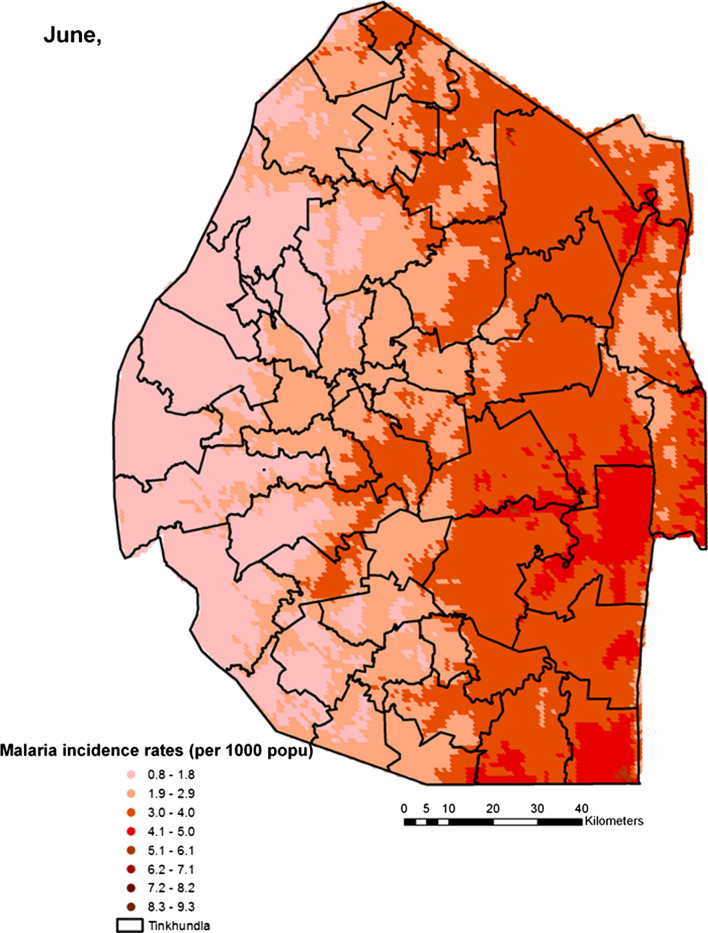
Malaria incidence during the month of June

Spatial heterogeneities of malaria incidence due to climatic and environmental factors were identified for each full transmission season in Eswatini. Monthly variations in malaria incidence made it possible to visualize months of low, moderate and high incidence rates in the country. High incidence rates were identified for the months of July to October (Figs. 4, 5, 6 and 7), moderate incidence rates in the months of November to February (Figs. 8, 9, 10 and 11) and low incidence rates in the months of March to June (Figs. 12, 13, 14 and 15). There was a positive association between temperature, rainfall and NDVI and an increase in malaria cases at polynomial lags of up to two months (8 weeks). For instance the current bi-week of (LST day[1]) was positively associated with malaria incidence [2.18 (BCI: 0.98–3.19)] while, the first lag or power $x$ ^1^ (LST day[2]) was negatively associated with malaria as cases decreased by − 2.63 (BCI: − 2.89– − 2.34).

Interestingly, third bi-week or power $x$ ^2^ (LST day[3]) and fourth bi-week or power $x$ ^3^ (LST day[4]) were positively associated with malaria cases with proportion 0.1 (BCI: 0.05–0.17) and proportion 0.22 (BCI: 0.22–0.2357) respectively. The increase in malaria cases changes in the fifth bi-week or power $x$ ^4^ (LST day[5]) where the proportion of cases begin to decrease. For night temperatures (LST night), the effects of the polynomial distributed lags was different in the sense that the current bi-week and the first lag were not associated with malaria incidence until the second bi-week or power $x$^2^ (LST night [3]) which was negatively associated with malaria at proportion − 0.12 (BCI: − 0.13– − 0.10). The third bi-week or power $x$^4^ was positively associated with malaria which changed in bi-week of power $x$^4^ where there was a negative association. On the other hand NDVI had less effect on malaria for the first two lags until power $x$^3^ (NDVI [4]) which showed increase in malaria cases with proportion 0.06359 (BCI: 0.05515–0.06808). Similarly to the first DLM model, malaria cases decreased with increasing rainfall as shown in power $x$^1^ (Rain[2]) and power $x$^3^ (Rain [4] with proportion -0.78 (BCI: − 0.95– − 0.19) and proportion − 0.01 (BCI: − 0.017– − 0.005) respectively. Interestingly rainfall was positively associated with malaria in power $x$^2^ (Rain [3]) with proportion 0.23 (BCI: 0.22–0.25). The results are summarized in Table 3 which shows the posterior probabilities of the polynomial DLM.

**Table 3:** Posterior estimates of polynomial distributed lags

Variable($x$ ^4^)	Mean	Sd	BCI: 2.5	Median	BCI: 97.5
LST day [1]	2.1800	0.6570	0.9857	2.3260	3.1900
LST day [2]	− 2.6310	0.1884	− 2.8900	− 2.6620	− 2.3410
LST day [3]	0.1064	0.0339	0.0561	0.0987	0.1741
LST day[4]	0.2284	0.0046	0.2206	0.2295	0.2357
LST day [5]	− 0.0292	0.0004	− 0.0300	− 0.0292	− 0.0288
LST night [1]	0.0062	0.3635	− 0.5658	0.1021	0.5285
LST night [2]	0.2757	0.2006	− 0.0097	0.2314	0.5429
LST night [3]	− 0.1240	0.0086	− 0.1372	− 0.1264	− 0.1077
LST night [4]	0.0287	0.0014	0.0266	0.0293	0.0308
LST night [5]	− 0.0027	0.0003	− 0.0033	− 0.0026	− 0.0022
NDVI [1]	− 0.3156	0.3682	− 0.9960	− 0.2119	0.2182
NDVI [2]	− 0.2407	0.1474	− 0.4127	− 0.2853	0.0081
NDVI [3]	0.0012	0.0162	− 0.0197	− 0.0024	0.0428
NDVI [4]	0.0636	0.0034	0.0552	0.0644	0.0681
NDVI [5]	− 0.0088	0.0002	− 0.0092	− 0.0088	− 0.0085
Rain [1]	0.4434	0.4889	− 0.9898	0.6221	0.9489
Rain [2]	− 0.7833	0.1917	− 0.9561	− 0.8864	− 0.1917
Rain [3]	0.2381	0.0102	0.2200	0.2379	0.2587
Rain [4]	− 0.0104	0.0024	− 0.0175	− 0.0102	− 0.0058
Rain [5]	− 0.0013	0.0003	− 0.0020	− 0.0013	− 0.0008

*Sd* standard deviation, *BCI* Bayesian credible interval

## Discussion and Conclusions

The influence of climatic and environmental factors on malaria risk had been well investigated however, this study is the first to produce monthly predictions of malaria incidence risk distribution using DLM in Eswatini. The maps produced in the current work depict a considerable month to month fluctuation in malaria incidence rates in the country and the best predictors in the DLM model included rainfall of the preceding month (Lag2), night temperature of the first and second preceding months. A similar result was found in a study by [38] where the best predictors including NDVI, mean maximum temperature and rainfall of the preceding month increased the number of malaria cases.

The current study provides useful information for timing and guiding deployment of malaria control measures as the country continues to fight sporadic cases. The dry season was associated with an increased number of malaria reported cases and this was not surprising as similar studies had already shown that persistent rainfall associated with the wet season have a tendency to wash out mosquito larvae thus hindering reproduction. For instance, a study by [39] found that increasing rainfall reduced malaria incidence in Nigeria. This then calls for intensification of surveillance efforts during the dry season or ideally immediately at the end of the wet season.

The results also showed that decrease in the amount of rainfall for over a two-month period was a precursor for cases in the next coming days. A study by [40] found strong positive correlations for malaria time series lagging zero to three months behind rainfall, and negative correlations were found for malaria time series lagging four to nine months behind rainfall. This study has also clearly demonstrated that polynomial distributed rainfall lags at least beyond two months were negatively correlated with malaria reported cases. Knowing this could enable the malaria control programme to be on alert and anticipate epidemics and astutely deploy the necessary prevention strategies. It was noted that the location of nearby imported cases was an important determinant of secondary infections and subsequently local transmission.

The findings presented in this work provide more critical considerations as well as an opportunity for the malaria programme to bolster its surveillance efforts and record the first elimination of the disease in its entire malaria history. While sporadic cases remain and importation continue to thwart and retard ongoing elimination efforts recent progress especially, the drastic reduction of malaria cases had shown that elimination was very much achievable and possible for the country.

The monthly incidence risk maps produced in this work could be useful for the control programme as they provided an explicit guide for resource optimization by showing the areas that need to be targeted with malaria intervention to achieve high impact. The maps could be used as a guide for timely monthly effective targeting and optimal deployment of resources for malaria prevention and response [41]. While high incidence risk was predominantly on the eastern lowlands of the country, its magnitude varied from month to month and this would be key to understanding the inter-annual variations and distributions of the disease. Previous analyses efforts were limited to seasonality in terms of wet and dry (summer and winter), our work had shown how malaria cases are distributed by month further unpacking the conventionally known seasonality thus, bringing more clarity and elucidating uncertainties in seasonality modeling [42]. Malaria transmission in Eswatini had been known to occur in the wet season of November to May with very fewer cases occurring in the dry season of June to October. This study has for the first time produced a new monthly pattern of malaria incidence rates comprising of three transmission seasons which were: July to October (high), November to February (moderate) and March to June (low). These identified seasons could be used to guide ongoing malaria surveillance efforts as the country pushes towards elimination. High malaria incidence rates were identified in the eastern part of the country especially in the Lowveld ecological zone which has higher average temperatures compared to the western part of the country which has higher elevation and lower average temperatures. This was followed by the Middleveld and the Lubombo Plateau ecological zones which have lower average temperatures and higher altitude compared to the Lowveld. The western part of the country which comprises of the Highveld zone had the lowest malaria incidence rates mainly due to high altitude and lower average temperatures. The high transmission season may be attributed to the fact that this is usually the period after the rainy season when mosquitoes may be able to lay eggs and breed compared to the rainy season when the eggs and larvae may be washed away [43]. The November month which marks the beginning of the moderate transmission season coincide with the beginning of the rainy season in Eswatini as the country approaches summer. Also, the highest amount of summer rainfall in the country is mostly received around February to March which in our study was identified as the low transmission season probably due to the excess runoff that flushes away mosquito larvae thus hindering breeding [44].

Day temperatures of the preceding first month were found to be important predictors for malaria incidence while the first monthly lag had a negative effect as it reduced malaria incidence. A study by [45] also found that monthly mean minimum temperature, mean maximum temperature, mean average temperature, were significantly and positively correlated with monthly malaria cases. This means that increased surveillance and vigilance would be needed at least after four consecutive weeks of high day temperatures especially during the identified transmission seasons. Vegetation was positively associated with malaria incidence after a fourth bi-week lag indicating that malaria incidence only increased after at least two months as a result of vegetation cover. Similarly, [46] also found that NDVI lagging by 1 and 2 months had a significant influence on malaria incidence. This is because thick shrubs and smaller plants can create or alter the surrounding microclimate in which mosquitoes can rest outdoor. Therefore, vegetation around a home is likely to be an important determinant of malaria breeding and even transmission [47].

The Bayesian geostatistical models developed in this study could be extended and applied in the development of rapid short-term and long-term forecasting algorithms that could assist the country with targeted prevention and response to effectively eliminate local transmission. We developed a polynomial DLM model which showed monthly distributions of malaria incidence rates, an important step for very low malaria endemic settings like Eswatini. This could help the NMP to understand the micro epidemiology of the disease in space and time and thereby target and optimally deploy malaria interventions in accordance with the severity of the observed malaria episodes. In order for the country to successfully eliminate malaria more scientifically based surveillance efforts need to be used. The current work provides a strategic guide for the ongoing malaria elimination efforts in Eswatini. The main limitation of this study was that the data used were not adequate in some months as cases drastically decreased at the height of the elimination efforts. This may have affected the temporal analyses in this study as some months had very few cases and made it difficult to accurately estimate the parameters. Also, the effects of climate change may alter the seasonal and monthly malaria incidence forecasts made in this study as the weather and environmental conditions changes.

## Data Availability

Data was obtained from the NMP Programme and permission was granted by the Programme Manager to use the data in the study*.*

## Abbreviations

ADDS: Africa data dissemination service
ASTER: Advanced spaceborne thermal emission and reflection radiometer
BCI: Bayesian credible interval
DEM: Digital elevation model
DLM: Distributed lag model
EA: Enumeration area
FEWS: Famine early warning systems
LST: Land surface temperature
MCMC: Markov chain Monte Carlo
MODIS: Moderate resolution imaging spectroradiometer
NDVI: Normalized difference vegetation index
NMP: National malaria programme
NMCP: National malaria control programme
